# Regulation of Asymmetric Division by Atypical Protein Kinase C Influences Early Specification of CD8^+^ T Lymphocyte Fates

**DOI:** 10.1038/srep19182

**Published:** 2016-01-14

**Authors:** Patrick J. Metz, Justine Lopez, Stephanie H. Kim, Kazunori Akimoto, Shigeo Ohno, John T. Chang

**Affiliations:** 1 Department of Medicine, University of California San Diego, La Jolla, CA 92093, USA; 2 Department of Molecular Biology, Yokohama City University School of Medicine, Kanazawa, Yokohama, Kanagawa Prefecture 236–0027, Japan

## Abstract

Naïve CD8^+^ T lymphocytes responding to microbial pathogens give rise to effector T cells that provide acute defense and memory T cells that provide long-lived immunity. Upon activation, CD8^+^ T lymphocytes can undergo asymmetric division, unequally distributing factors to the nascent daughter cells that influence their eventual fate towards the effector or memory lineages. Individual loss of either atypical protein kinase C (aPKC) isoform, PKCζ or PKCλ/ι, partially impairs asymmetric divisions and increases CD8^+^ T lymphocyte differentiation toward a long-lived effector fate at the expense of memory T cell formation. Here, we show that deletion of both aPKC isoforms resulted in a deficit in asymmetric divisions, increasing the proportion of daughter cells that inherit high amounts of effector fate-associated molecules, IL-2Rα, T-bet, IFNγR, and interferon regulatory factor 4 (IRF4). However, unlike CD8^+^ T cells deficient in only one aPKC isoform, complete loss of aPKC unexpectedly increased CD8^+^ T cell differentiation toward a short-lived, terminal effector fate, as evidenced by increased rates of apoptosis and decreased expression of Eomes and Bcl2 early during the immune response. Together, these results provide evidence for an important role for asymmetric division in CD8^+^ T lymphocyte fate specification by regulating the balance between effector and memory precursors at the initiation of the adaptive immune response.

A single naïve CD8^+^ T lymphocyte can give rise to both effector and memory T cell subsets during a microbial infection^1^,^2^. Effector T cells provide acute host defense early during the immune response and rapidly undergo apoptosis following clearance of the infection^3^. Despite this propensity to undergo apoptosis, some effector T cells can survive into the memory phase of an adaptive immune response^4^,^5^. These long-lived effector cells exert a potent protective response against re-infection but maintain a poor recall response^4^,^5^. Two populations, effector memory T (T_EM_) cells and central memory T (T_CM_) cells, comprise the circulating memory lymphocyte pool that persists long-term following an acute infection. T_EM_ cells circulate through the peripheral tissues and provide immediate effector function upon rechallenge, whereas T_CM_ cells maintain a capacity for robust proliferation upon antigen re-encounter^6^. While production of a heterogeneous adaptive immune response is necessary for robust protection against microbial infection, the ontology of these various CD8^+^ T lymphocytes subsets remains poorly understood.

Differentiation into the effector and memory T lymphocyte subsets depends on signaling through key cytokine receptors and the expression of important transcription factors^7^. IL-2 and IFNγ are two such cytokines, and signals downstream of the receptors for either cytokine, IL-2Rα and IFNγR, reinforce differentiation into the effector fates via upregulation of the transcription factor, T-bet^8^,^9^,^10^,^11^, an essential transcription factor for terminal effector cell formation^12^. Conversely, a closely related T-box protein, Eomesodermin (Eomes), is thought to be responsible for controlling memory differentiation, in part, by upregulating anti-apoptotic molecules, such as B cell lymphoma 2 (Bcl2)^13^, that prevent premature cell death as T lymphocytes progress through the immune response^14^. Recently, interferon regulatory factor 4 (IRF4) has been shown to be important for differentiation of terminal effector T cells^15^,^16^,^17^. IRF4 has been found to negatively regulate the expression of Eomes^18^, suggesting that differentiation into the memory T cell subsets may require the exclusion or loss of effector fate-associated factors.

Upon activation with antigen, a CD8^+^ T lymphocyte can undergo asymmetric division, whereby important cell components and fate determinants are unequally distributed between the two nascent daughter cells as the cell divides^19^,^20^,^21^,^22^. Key effector fate-associated factors, including T-bet, IL-2Rα, and IFNγR, are among those proteins that are asymmetrically partitioned during the first division^19^,^20^,^21^, thereby contributing to divergent pathways of differentiation into the effector or memory T lymphocyte fates^19^,^23^. The evolutionarily conserved polarity protein, atypical protein kinase C (aPKC), regulates asymmetric division in model organisms^24^,^25^,^26^, and our previous work has shown that the aPKC isoforms, PKCζ and PKCλ/ι, individually control asymmetric divisions by CD8^+^ T lymphocytes^23^. The absence of PKCζ or PKCλ/ι resulted in a modest increase in the symmetric distribution of key effector fate-associated molecules during the first division by activated CD8^+^ T lymphocytes, which subsequently increased differentiation toward the long-lived effector fate at the expense of the memory fates^23^. However, it remained unknown whether one isoform of aPKC could compensate for the absence of the other. Furthermore, it remained unknown whether the complete absence of aPKC would result in a more profound defect in asymmetric divisions and a similar or unique consequence on the acquisition of effector and memory T cell fates.

To understand the full effect of aPKC in regulating asymmetric division and fate specification of CD8^+^ T lymphocytes, we therefore generated mice with a T cell-specific deletion of both aPKC isoforms. Here, we show that complete loss of aPKC resulted in a more profound increase in the proportion of daughter cells predisposed toward the effector fates, a consequence of defective asymmetric divisions by activated CD8^+^ T lymphocytes, which caused more daughter cells to acquire high amounts of effector fate-associated molecules. However, unlike CD8^+^ T cells deficient only in one aPKC isoform, complete loss of aPKC increased differentiation into the terminal effector cell fate at the expense of long-lived effector and memory CD8^+^ T lymphocyte formation. Moreover, aPKC-deficient CD8^+^ T cells exhibited increased rates of apoptosis and decreased expression of Eomes and Bcl2 early during the immune response, suggesting that expression of these two factors is necessary for survival of effector-fated precursor cells. Together, these findings indicate that PKCζ and PKCλ/ι may have important roles in regulating asymmetric division, thereby mediating early CD8^+^ T lymphocyte fate specification by regulating the balance between effector and memory precursor cells.

## Results

### Loss of aPKC does not affect CD8^+^ T lymphocyte activation

**Figure 1:**
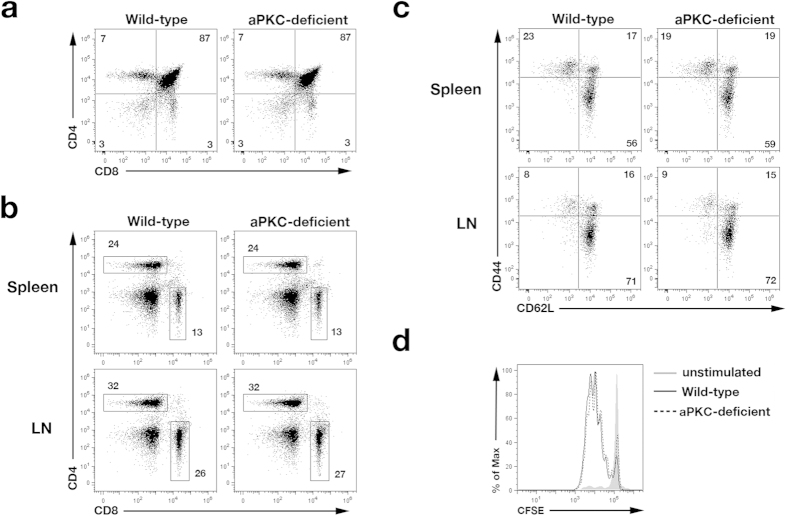
T lymphocytes in aPKC-deficient mice develop normally. (**a**) Frequencies of double-negative, double-positive, and single-positive CD4 and CD8 T cells from thymi of wild-type and aPKC-deficient mice. (**b**) Frequencies of CD4^+^ and CD8^+^ T cells from spleens and lymph nodes of wild-type and aPKC-deficient mice. (**c**) Frequencies of naïve CD8^+^ T cells (CD62L^hi^CD44^lo^) and effector memory or central memory CD8^+^ T cells (CD62L^lo^CD44^hi^ and CD62L^hi^CD44^hi^, respectively) from spleens and lymph nodes of wild-type and aPKC-deficient mice. (**d**) CFSE dilution of unstimulated (gray filled), wild-type (solid black), and aPKC-deficient (dashed black) CD8^+^ OT-I TCR transgenic T cells after 3 days *in vitro* culture with IL-2 and irradiated, T cell-depleted splenocytes pulsed with 1μM ovalbumin peptide. Data are representative of three experiments.

To study the role of both aPKC isoforms in CD8^+^ T lymphocyte asymmetric division and differentiation, we generated *Prkcz*^fl/fl^*Prkci*^fl/fl^ (wild-type) and *Cd4*^Cre^*Prkcz*^fl/fl^*Prkci*^fl/fl^ (hereafter referred to as ‘aPKC-deficient’) mice. To assess T cell development and homeostasis, thymus, spleen, and lymph nodes from wild-type and aPKC-deficient mice were analyzed. Atypical PKC-deficient thymi contained similar percentages of double-negative, double-positive, and single-positive CD4 and CD8 T lymphocyte populations compared to those from wild-type control mice (Fig. 1a), and we observed similar percentages of CD4^+^ and CD8^+^ T cells in the spleen and lymph nodes (Fig. 1b). Previous activation of CD8^+^ T lymphocytes to environmental antigens, assessed by staining for CD44 and CD62L, was also similar between wild-type and aPKC-deficient mice (Fig. 1c).

To assess the ability of aPKC-deficient CD8^+^ T cells to respond to antigen following dendritic cell engagement, we bred wild-type and aPKC-deficient mice to OT-I TCR transgenic mice to generate mice with CD8^+^ T cells that recognize amino acids 257–264 (SIINFEKL) of chicken ovalbumin^27^. Expression of the OT-I TCR did not affect T cell development in aPKC-deficient mice (data not shown). Purified wild-type and aPKC-deficient CD8^+^ T cells were labeled with CFSE and cultured *in vitro* with ovalbumin peptide-pulsed splenocytes. We observed that aPKC-deficient CD8^+^ T lymphocytes proliferated comparably to wild-type controls (Fig. 1d), suggesting that antigen-induced activation of naïve CD8^+^ T cells is not affected by the loss of aPKC. Together, these data show that loss of aPKC does not alter development or activation of naïve CD8^+^ T cells.

### aPKC regulates asymmetric division and controls the ratio of effector-fated and memory-fated T cells

**Figure 2:**
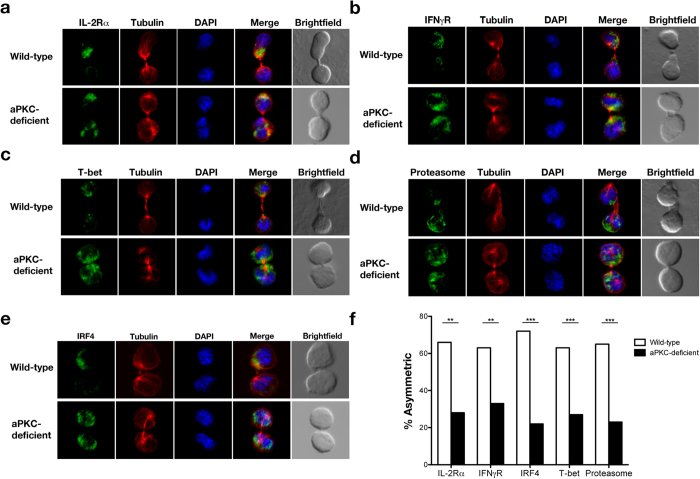
Loss of aPKC impairs asymmetric segregation of effector fate-associated factors during the first division of an activated CD8^+^ T cell. Confocal microscopy of (**a**) IL-2Rα, (**b**) IFNγR, (**c**) T-bet, (**d**) proteasome, or (**e**) IRF4 (green), β-tubulin (red), and DNA (blue; stained with the DNA-intercalating dye DAPI) in sorted wild-type or aPKC-deficient OT-I CD8^+^ T cells undergoing their first division after adoptive transfer into Lm-OVA infected recipient mice. (**f**) Incidence of asymmetric protein localization from T cells shown in (**a–e**). The number of dividing cells from two experiments is indicated in parenthesis as follows (wild-type, aPKC-deficient): IL-2Rα (44, 32), IFNγR (38, 64), T-bet (59, 63), proteasome (54, 60), and IRF4 (18, 34). **P < 0.01, ***P < 0.001 (one-tailed Fisher’s exact test).

During an asymmetric division by an activated naïve CD8^+^ T lymphocyte, important effector fate-associated factors, such as IFNγR and IL-2Rα, localize to the daughter cell ‘proximal’ to the antigen-presenting cell^19^,^21^. In addition, asymmetric distribution of the proteasome degradation machinery causes preferential destruction of the effector fate-associated transcription factor, T-bet, in the daughter cell ‘distal’ to the immune synapse^20^. Our previous work suggested that the aPKC isoforms individually regulate asymmetric division, functioning to exclude effector fate-associated factors from the distal daughter cell^23^. To investigate the effect of complete aPKC deficiency in the localization of these proteins, we used our previously established *in vivo* method to isolate activated naïve CD8^+^ T cells undergoing their first division in response to a microbial pathogen^21^. CFSE-labeled wild-type or aPKC-deficient OT-I CD8^+^ T cells were adoptively transferred into wild-type recipients, which had been infected with recombinant *Listeria monocytogenes* expressing ovalbumin (Lm-OVA) 24 hours prior. At 36 hours post-transfer, undivided donor CD8^+^ T lymphocytes were sorted and examined by confocal microscopy. We observed a substantial decrease in the ability of aPKC-deficient CD8^+^ T cells to maintain the unequal localization of IL-2Rα, IFNγR, T-bet, and the proteasome through the completion of mitosis (Fig. 2a–d,f). Moreover, examination of activated wild-type CD8^+^ T cells undergoing their first division revealed a high rate of asymmetric localization of IRF4, a recently identified factor regulating terminal effector differentiation^15^,^16^,^17^, whereas examination of activated aPKC-deficient CD8^+^ T cells showed a marked decrease in the asymmetric distribution of IRF4 through cytokinesis (Fig. 2e,f).

**Figure 3:**
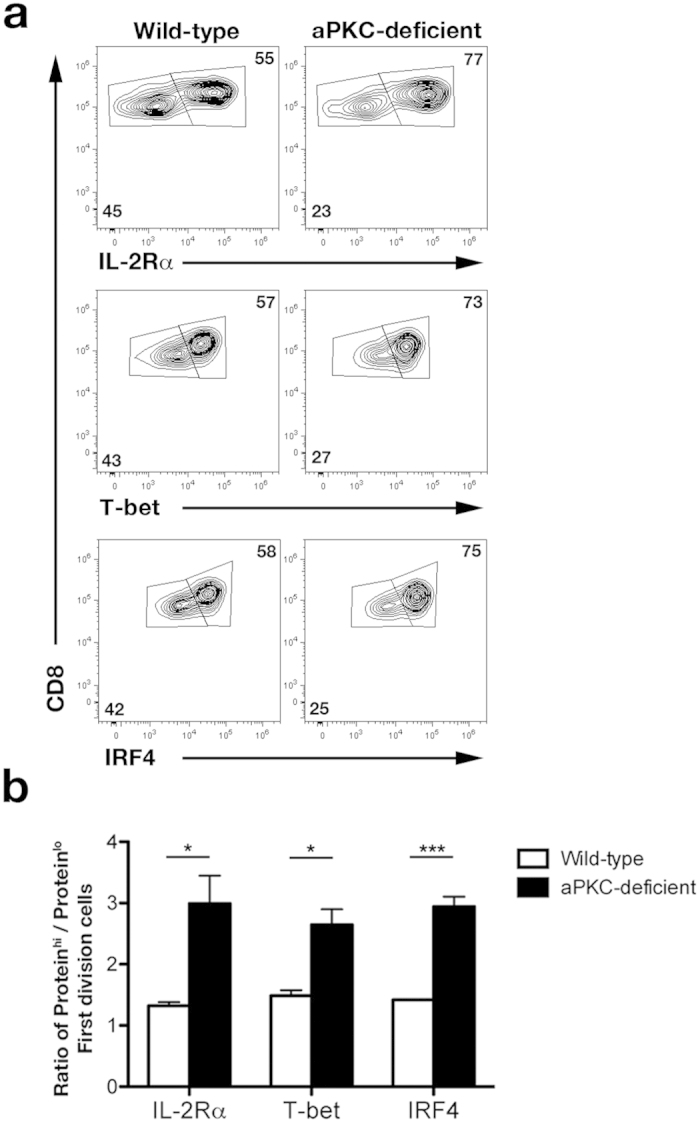
aPKC regulates the balance of pre-effector and pre-memory first division cells *in vivo*. (**a**) Expression of IL-2Rα, T-bet, and IRF4 by wild-type and aPKC-deficient first division OT-I CD8^+^ T cells after adoptive transfer into recipient mice and subsequent infection with Lm-OVA. (**b**) Ratios of high and low expression of the indicated protein from first division OT-I CD8^+^ T cells in (**a**); bars represent mean ± SEM; n = 3/group; *P < 0.05, ***P < 0.001 (two-tailed unpaired t-test). Data are representative of two experiments.

This defect in asymmetric distribution of effector-associated molecules in aPKC-deficient cells that had undergone their first division raised the possibility that these cells might be predisposed towards the effector fates. Our recent work has suggested that CD8^+^ T cells that have undergone their first division exhibit a gene expression signature predictive of their eventual fate as effector or memory T lymphocytes^19^. Putative ‘pre-effector’ cells, characterized by high expression of IL-2Rα following the first division, can give rise to the terminal effector cell fate, whereas putative ‘pre-memory’ cells, characterized by low expression of IL-2Rα following the first division, can further diverge to give rise to the T_EM_ and T_CM_ cell fates^19^. We therefore examined the expression of IL-2Rα and other effector-associated molecules by wild-type and aPKC-deficient first division cells. Compared to wild-type controls, we observed a 2.5- to 3-fold increased proportion of aPKC-deficient CD8^+^ T lymphocytes that not only expressed high levels of IL-2Rα but also high levels of T-bet and IRF4 (Fig. 3). This finding, compared to the 1.5- to 2-fold alteration previously observed with first division CD8^+^ T lymphocytes deficient in only one isoform of aPKC^23^, suggests that loss of both aPKC isoforms may have a greater influence on pre-effector and pre-memory fate specification. One possible explanation for this difference, although not formally tested here, is that one aPKC isoform may compensate in the absence of the other. Together, these results suggest that disruption of asymmetric divisions, as a result of aPKC deficiency, promotes the generation of cells predisposed towards the effector differentiation pathway.

### Complete deficiency of aPKC promotes differentiation towards the terminal effector fate early during the immune response

**Figure 4:**
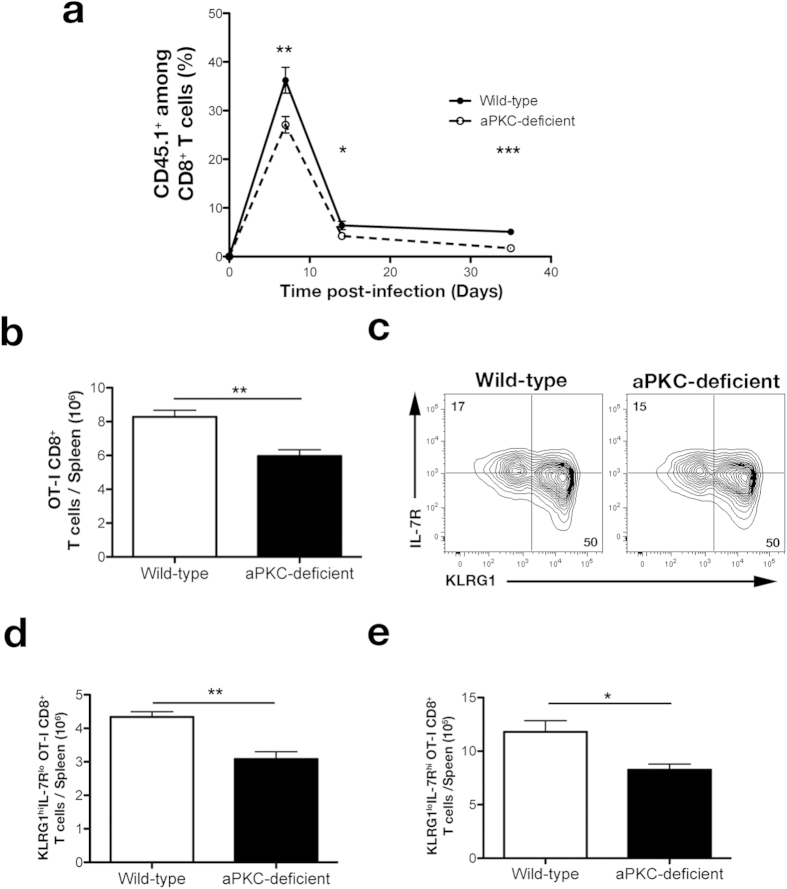
aPKC-deficient CD8^+^ T cells give rise to an impaired adaptive immune response at day 7 post-infection. (**a**) Percentages of CD45.1^+^ cells of CD8^+^ T cells on day 7, 14, and 35 post-infection in the blood of mice that received 5 × 10^3^ wild-type or aPKC-deficient OT-I CD45.1^+^CD8^+^ T cells and were infected with Lm-OVA; points represent mean ± SEM; n ≥ 4/group. (**b**) Total number of OT-I CD8^+^ T cells on day 7 post-infection in the spleens of mice that received 5 × 10^3^ wild-type or aPKC-deficient OT-I CD45.1^+^CD8^+^ T cells and were subsequently infected with Lm-OVA; bars represent the mean ± SEM; n = 3–4/group. (**c**) Expression of KLRG1 and IL-7R by CD45.1^+^CD8^+^ T cells in the spleen on day 7 post-infection. (**d,e**) Total number of (**d**) KLRG1^hi^IL-7R^lo^ effector, and (**e**) KLRG1^lo^IL-7R^hi^ putative memory precursor CD8^+^ T cells on day 7 post-infection in the spleens of mice that received 5 × 10^3^ wild-type or aPKC-deficient OT-I CD45.1^+^CD8^+^ T cells and were subsequently infected with Lm-OVA; bars represent the mean ± SEM; n = 3–4/group. *P < 0.05, **P < 0.01 and ***P < 0.001 (two-tailed unpaired t-test). Data are representative of three experiments.

Our previous work demonstrated that alterations to the ratio of pre-effector to pre-memory cells, as a result of defective asymmetric divisions, increased CD8^+^ T lymphocyte differentiation toward the long-lived effector fate at the expense of the memory fates following infection^23^. The finding of a more pronounced increase in the ratio of pre-effector to pre-memory precursor cells by CD8^+^ T cells deficient in both aPKC isoforms, compared to cells deficient in only one isoform, suggested that aPKC-deficient cells might experience a more profound increase in differentiation toward the long-lived effector cell fate. To investigate the functional effect that complete loss of aPKC has on the CD8^+^ T cell response, we adoptively transferred wild-type or aPKC-deficient OT-I CD45.1^+^CD8^+^ T cells into separate CD45.2^+^ wild-type recipients, which were infected 24 hours later with Lm-OVA. Unlike CD8^+^ T lymphocytes deficient in only one aPKC isoform, which increased differentiation into the long-lived effector fate without altering the kinetics of the immune response^23^, complete loss of aPKC resulted in a 20% reduction in the percentage and number of CD8^+^ T lymphocytes at 7 days post-infection compared to wild-type CD8^+^ T lymphocytes (Fig. 4a,b). This small reduction in the percentage of CD8^+^ T lymphocytes observed at day 7 post-infection in mice that received aPKC-deficient OT-I T cells progressed to a 66% reduction by day 35 post-infection (Fig. 4a). This progressive decline of aPKC-deficient CD8^+^ T lymphocytes through the course of the adaptive immune response suggested an increased presence of terminal effector cells within mice that received aPKC-deficient T cells. High expression of the lectin-like receptor, KLRG1, is thought to distinguish cells of the terminal effector fate at day 7 post-infection, whereas high expression of IL-7R is thought to distinguish memory precursor cells^28^; however, examination of these markers revealed no change in the percentage of aPKC-deficient T cells expressing high levels of either KLRG1 or IL-7R compared to wild-type control cells (Fig. 4c). Moreover, decreased numbers of both putative effector (KLRG1^hi^IL-7R^lo^) and memory precursor cells (KLRG1^lo^IL-7R^hi^) were observed in the spleens of mice that received aPKC-deficient T cells at day 7 post-infection (Fig. 4d,e).

**Figure 5:**
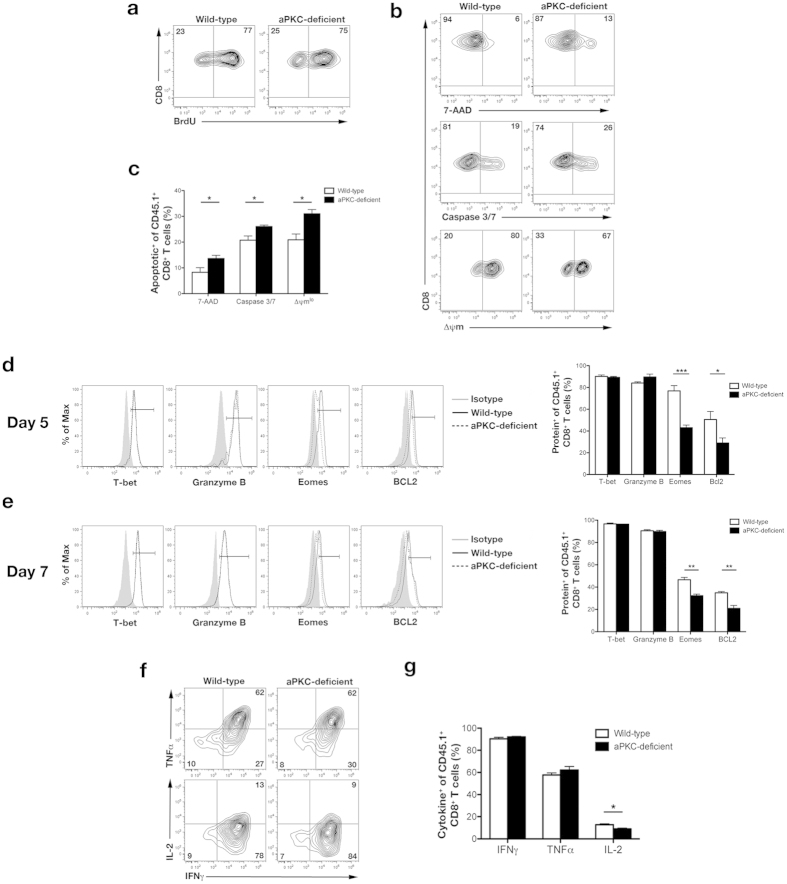
Deficiency of aPKC increases differentiation into the terminal effector fate early during the immune response. (**a**) BrdU incorporation after a 1 hour pulse-chase by wild-type or aPKC-deficient OT-I CD8^+^ T cells on day 5 post-infection in the spleens of mice that received 5 × 10^3^ wild-type or aPKC-deficient OT-I CD45.1^+^CD8^+^ T cells and were infected with Lm-OVA. (**b**) 7-AAD uptake (top), active caspase-3/-7 (middle), and mitochondrial membrane potential (Δψm) (bottom) of wild-type or aPKC-deficient OT-I CD8^+^ T cells on day 5 post-infection in the spleens of mice that received cells as in (**a**). (**c**) Frequencies of apoptotic (7-AAD^+^, Caspase 3/7^+^, or Δψm^lo^) wild-type or aPKC-deficient OT-I CD8^+^ T cells from mice shown in (b). Bars represent mean ± SEM, n = 4/group. (**d,e**) *Left,* expression of T-bet, Granzyme B, Eomes, and Bcl2 by wild-type (solid black) or aPKC-deficient (dashed black) CD45.1^+^CD8^+^ T cells on days (**d**) 5 or (**e**) 7 post-infection in the spleens of mice as in (**a**). Isotype controls (gray filled) are shown with the associated gates used to measure the percentage of cells expressing the indicated protein. *Right,* frequencies of expression of T-bet, Granzyme B, Eomes, and Bcl2 from mice shown on the left. Bars represent mean ± SEM, n = 3–4/group. (**f**) Expression of IFNγ, TNFα, and IL-2 by wild-type or aPKC-deficient OT-I CD45.1^+^CD8^+^ T cells following restimulation *ex vivo* for 6 hours with ovalbumin peptide on day 7 post-infection. (**g**) Frequencies of expression of IFNγ, TNFα, or IL-2 from mice shown in (**f**). Bars represent mean ± SEM, n = 3–4/group. *P < 0.05, **P < 0.01 and ***P < 0.001 (two-tailed unpaired t-test). Data are representative of (**a**) two or (**b–g**) three experiments.

While the differentiation patterns of aPKC-deficient CD8^+^ T lymphocytes appeared to be unchanged based on the expression of putative phenotypic markers early during the immune response, alterations to the kinetics of the aPKC-deficient immune response suggested that the aPKC isoforms had an important role in regulating the CD8^+^ T lymphocyte response. The magnitude of the peak of the immune response is controlled by the rates of proliferation and cell death^29^. The decreased numbers of aPKC-deficient cells at day 7 post-infection, therefore, suggested that aPKC-deficient cells would exhibit altered rates of proliferation or cell death relative to wild-type cells prior to day 7 post-infection. To understand why aPKC-deficient CD8^+^ T lymphocytes would give rise to reduced numbers of effector cells in addition to the expected reduction in memory precursor cells, we assessed proliferation and apoptosis at day 5 post-infection. Although CFSE dilution was normal following activation *in vitro* (Fig. 1d), we hypothesized that aPKC-deficient T cells may not have maintained a normal rate of division leading up to the peak of the immune response. To address this possibility, we performed a bromodeoxyuridine (BrdU) pulse-chase experiment, in which actively dividing cells are able to incorporate BrdU into replicating DNA during S phase^30^. Following a 1-hour pulse-chase at 5 days post-infection, we observed a similar percentage of aPKC-deficient cells that were BrdU^+^ compared to wild-type control cells (Fig. 5a), suggesting that aPKC-deficient cells did not have a defect in their ability to divide during the immune response. To assess the percentage of cells undergoing apoptosis, we used 7-AAD uptake^31^, cleaved caspase-3/-7^32^, and maintenance of mitochondrial membrane potential (Δψm)^33^ as indicators of cells that were primed for or undergoing cell death. Examination of CD8^+^ T lymphocytes at 5 days post-infection using these three metrics revealed that aPKC-deficient cells were more likely to undergo apoptosis compared to wild-type cells (Fig. 5b,c).

As apoptosis is one hallmark of the terminal effector fate^3^, these findings suggested that despite a decrease in the number of cells expressing putative phenotypic markers, aPKC-deficient CD8^+^ T lymphocyte differentiation had, in fact, favored the terminal effector fate. Terminal effector T cells are characterized by high levels of T-bet and the cytotoxic molecule, Granzyme B, and low levels of the memory fate-associated molecule, Eomes, and the pro-survival factor, Bcl2^12^,^34^,^35^,^36^,^37^. Furthermore, terminal effector cells have been shown to lack the capacity to produce IL-2^12^. Examination of T-bet and Granzyme B revealed no differences between wild-type and aPKC-deficient CD8^+^ T cells; however, we observed that aPKC-deficient CD8^+^ T lymphocytes displayed decreased expression of Eomes and Bcl2 compared to wild-type control cells at both 5 and 7 days post-infection (Fig. 5d,e). Following restimulation at 7 days post-infection, a similar percentage of aPKC-deficient CD8^+^ T lymphocytes produced IFNγ and TNFα compared to wild-type control cells; however, the percentage of aPKC-deficient cells that were capable of producing IL-2 was reduced (Fig. 5f,g). Together, these data suggest that complete loss of aPKC increased CD8^+^ T cell differentiation towards the terminal effector fate, thereby resulting in accelerated death of effector cells and reducing the total number of CD8^+^ T lymphocytes present at the peak of the immune response.

### aPKC influences specification of memory CD8^+^ T lymphocyte fates

**Figure 6:**
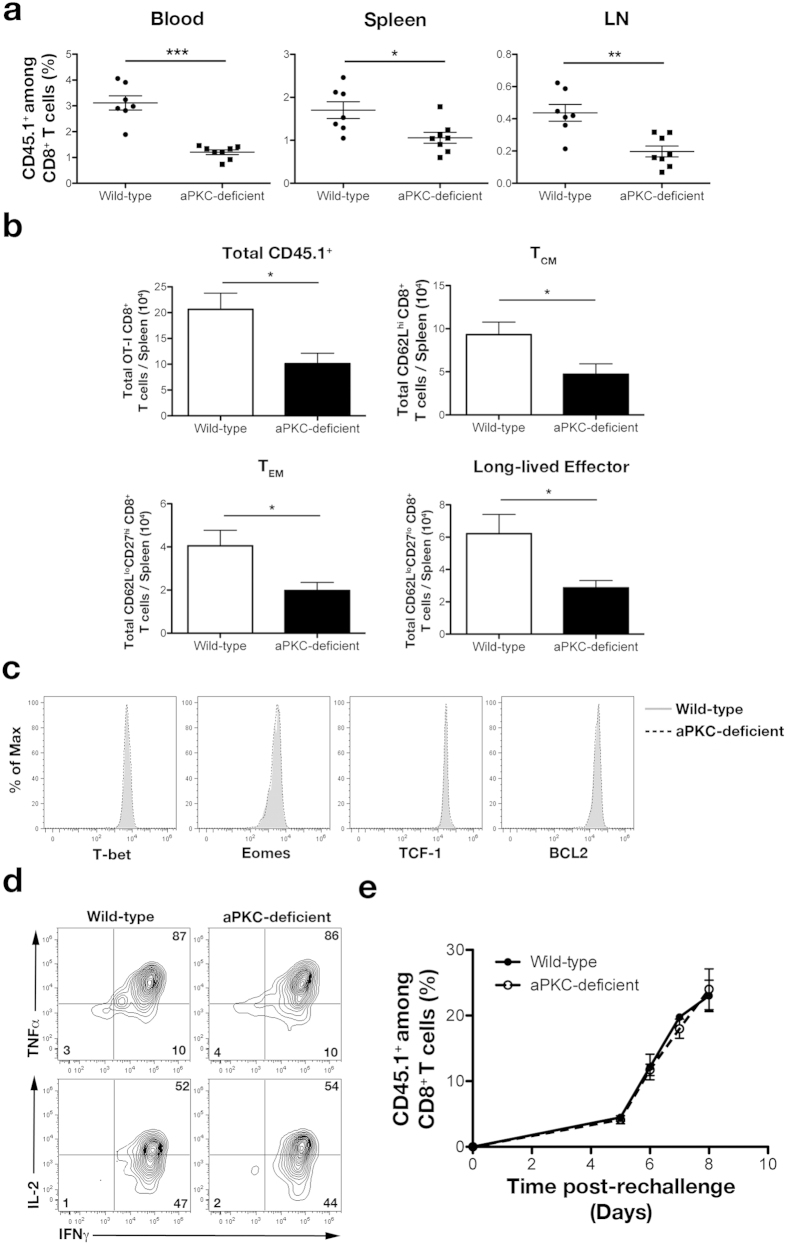
aPKC regulates long-lived CD8^+^ T cell differentiation. (**a**) Frequencies of CD45.1^+^CD8^+^ T cells in the blood (left), spleen (middle), and lymph nodes (right) on day 50 post-infection; each point represents an individual mouse and lines indicate the mean ± SEM; n = 7–8/group. (**b**) Total number of OT-I (left), CD62L^hi^ T_CM_ (middle), and CD62L^lo^ T_EM_ (right) CD45.1^+^CD8^+^ T cells in the spleen on day 50 post-infection; bars represent mean ± SEM; n = 7–8/group. (**c**) Expression of T-bet, Eomes, Tcf-1, and Bcl2 in wild-type (gray filled) and aPKC-deficient (dashed black) CD45.1^+^CD8^+^ cells in the spleen on day 50 post-infection. (**d**) Expression of IFNγ, TNFα, and IL-2 by wild-type or aPKC-deficient OT-I CD45.1^+^CD8^+^ T cells following restimulation *ex vivo* for 6 hours with ovalbumin peptide on day 50 post-infection. (**e**) Frequencies of CD45.1^+^CD8^+^ T cells on days 5, 6, 7, and 8 in the blood of mice that received 10^4^ wild-type or aPKC-deficient CD45.1^+^CD8^+^ T cells on day 50 post-infection and were subsequently challenged with 10^5^ CFU Lm-OVA. Points represent mean ± SEM; n = 4/group. For (**a,b**) *P < 0.05, **P < 0.01, and ***P < 0.001 (two-tailed unpaired t-test). Data are representative of three experiments.

Because aPKC-deficient T cell differentiation appeared to be increased toward the terminal effector cell fate, we predicted that differentiation of aPKC-deficient CD8^+^ T lymphocytes into the long-lived effector and memory cell fates would be correspondingly decreased compared to wild-type cells. Indeed, at day 50 post-infection, we observed that mice that received aPKC-deficient CD8^+^ T lymphocytes displayed a 50–70% reduction in the percentages of CD8^+^ T cells present in the blood, spleen, and lymph nodes compared to mice that received wild-type cells (Fig. 6a). Additionally, mice that received aPKC-deficient CD8^+^ T lymphocytes exhibited a decrease in the total number of OT-I cells and the total number of long-lived effector, T_EM_, and T_CM_ CD8^+^ T cells present in the spleen (Fig. 6b).

Although the percentage and number of aPKC-deficient CD8^+^ T cells were decreased, expression of T-bet, Eomes, Bcl2, and the memory fate-associated transcription factor, T cell factor 1 (Tcf-1)^38^, was similar within wild-type and aPKC-deficient CD8^+^ T lymphocytes (Fig 6c). Additionally, the ability to produce cytokines IFNγ, TNFα, and IL-2 was unaffected in aPKC-deficient CD8^+^ T lymphocytes at 50 days post-infection (Fig. 6d). Upon adoptive transfer of equal numbers of long-lived CD45.1^+^ T cells into wild-type recipients followed by subsequent challenge, aPKC-deficient CD8^+^ T lymphocytes were equally capable of proliferating in response to a secondary challenge compared to wild-type control cells (Fig. 6e). Together, these results indicate that aPKC is required for optimal formation of the long-lived effector and memory CD8^+^ T lymphocyte compartments; however, once formed, long-lived aPKC-deficient T cells function normally on a per cell basis.

### aPKC is required selectively at the first division for fate specification

**Figure 7:**
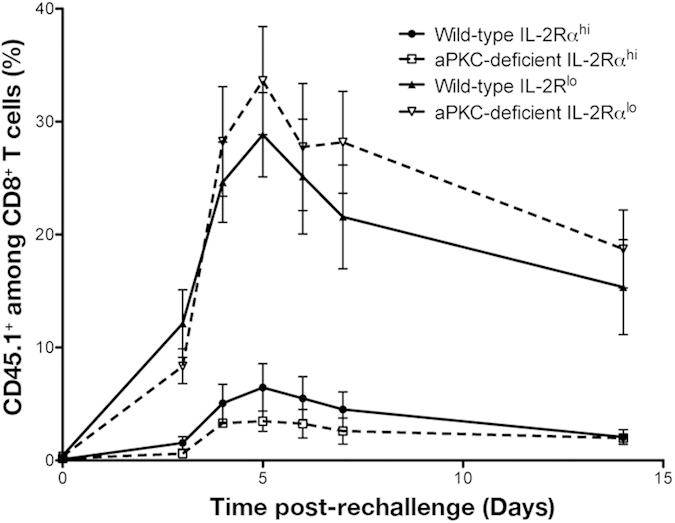
aPKC acts selectively at the first division by activated naïve CD8^+^ T lymphocytes. Population expansion of CD45.1^+^CD8^+^ T cells on days 3, 4, 5, 6, 7, and 14 post-rechallenge in blood obtained from Lm-OVA infected CD45.2^+^ mice that received 500 sort-purified wild-type IL-2Rα^lo^ (closed triangles), wild-type IL-2Rα^hi^ (closed circles), aPKC-deficient IL-2Rα^lo^ (open triangles), or aPKC-deficient IL-2Rα^hi^ (open squares) first division CD45.1^+^CD8^+^ OT-I T cells on day 2 post-infection and were rechallenged with 10^5^ CFU Lm-OVA on day 50 post-transfer; points represent mean ± SEM; n = 3–4/group. Data are representative of two experiments.

While complete loss of aPKC resulted in a substantial loss of asymmetry during the first cell division (Figs 2 and 3), leading to enhanced differentiation into the terminal effector cell fate (Figs 4 and 5) and impaired generation of long-lived effector and memory lymphocytes (Fig. 6), it remained possible that aPKC might also have a functional role after the first division, particularly in preventing apoptosis of pre-effector CD8^+^ T lymphocytes. To address this possibility, we adoptively transferred CFSE-labeled wild-type or aPKC-deficient OT-I CD45.1^+^ CD8^+^ T cells into CD45.2^+^ wild-type recipients, which were infected 24 hours later with Lm-OVA. First division IL-2Rα^hi^ pre-effector or IL-2Rα^lo^ pre-memory wild-type or aPKC-deficient cells were sorted and then adoptively transferred into infection-matched recipients, followed by challenge 50 days post-transfer. IL-2Rα^lo^ pre-memory cells of either genotype proliferated robustly in response to rechallenge, whereas both wild-type and aPKC-deficient IL-2Rα^hi^ pre-effector cells failed to do so (Fig. 7). Moreover, aPKC-deficient pre-effector cells, despite failing to undergo robust proliferation, were nonetheless present at similar frequencies as their wild-type counterparts (Fig. 7). Because aPKC-deficient pre-memory and pre-effector cells respond similarly to secondary rechallenge as their respective wild-type controls, these data suggest that complete loss of aPKC does not inherently increase cell death and further indicate that aPKC is dispensable in CD8^+^ T lymphocytes once cells have acquired differential amounts of IL-2Rα. Instead, aPKC appears to play a critical role during the first CD8^+^ T cell division by regulating the balance of effector- and memory-fated precursor cells that arise via asymmetric distribution of important effector fate-associated molecules. Taken together, our findings indicate that aPKC acts during the first division by an activated CD8^+^ T lymphocyte to regulate asymmetric division, thereby influencing early cell fate specification and generation of an optimal adaptive immune response.

## Discussion

Balancing the numbers of effector and memory T lymphocytes is essential for generating an optimal adaptive immune response that can provide acute and long-term protection against microbial pathogens^39^. Our work here suggests that asymmetric division by activated naïve CD8^+^ T lymphocytes is an important first step in regulating effector and memory fate determination. Disruption of asymmetric divisions, via deletion of aPKC, generated more first division cells that inherit key effector fate-associated factors, such as T-bet and IRF4. Increased acquisition of effector fate-associated factors predisposed more aPKC-deficient cells toward the effector differentiation pathway, thereby generating more terminal effector cells, evidenced by the increased rates of apoptosis early during the immune response. Correspondingly, pre-effector cells failed to optimally populate the long-lived effector pool, and the decrease in first division cells predisposed toward the memory fates resulted in a marked decrease in the formation of T_EM_ and T_CM_ cell populations into the late phase of the adaptive immune response.

Disruption of asymmetric divisions increased the proportion of aPKC-deficient first division cells that expressed high levels of effector fate-associated factors. In contrast to asymmetric division, which yields one daughter cell with high levels of such factors and the other daughter cell with low levels^21^, a symmetric division might be predicted to result in two daughter cells with intermediate levels. However, the levels of expression of T-bet, IRF4, and IL-2Rα in aPKC-deficient pre-effector cells were high, similar to the high levels expressed by wild-type pre-effector cells, rather than being expressed at intermediate levels. One possible explanation for this unexpected result pertains to feed-forward mechanisms that some effector fate-associated factors, such as T-bet and IFNγR, utilize to promote their own continued upregulation^9^,^40^. Symmetric distribution of effector fate-associated molecules, such as T-bet, may thus initially result in intermediate levels of protein, levels that may be sufficient to initiate these feed-forward mechanisms, thereby allowing protein levels in both aPKC-deficient daughter cells to increase to high levels of expression. Such a mechanism might underlie the increased proportion of aPKC-deficient pre-effector precursor cells present after the first division, thereby resulting in increased differentiation of terminal effector T cells.

Another unexpected finding was the observation that complete loss of aPKC increased differentiation into the terminal effector cell fate, whereas individual loss of either PKCζ or PKCλ/ι increased differentiation into the long-lived effector fate^23^. In both instances, impaired asymmetric divisions increased specification of the pre-effector fate following the first division; however, complete loss of aPKC altered the balance of pre-effector to pre-memory cells at a 2.5- to 3-fold rate compared to the 1.5- to 2-fold rate of single knockout cells^23^. Furthermore, aPKC-deficient CD8^+^ T lymphocytes displayed decreased levels of expression of Eomes and Bcl2 early during the immune response whereas single knockout cells did not^23^, which suggests a possible divergence between the terminal and long-lived effector fates that may hinge on the maintenance or upregulation of both factors. Because cytokine signals are important for the expression of effector and memory associated transcription factors^41^, it is possible that the additional increase in pre-effector cells, or further reduction in pre-memory cells, resulting from complete loss of aPKC may have subsequently altered the ensuing inflammatory signals received by daughter cells as they progressed into fully mature fates. For instance, IL-2 signaling may be required for Eomes upregulation^11^ and T cell survival^42^. Because IL-2 is more likely to be produced by cells predisposed toward the memory fates^12^, the further reduction in aPKC-deficient pre-memory cells may have resulted in less IL-2 production early during the immune response, at a level below the threshold necessary for optimal survival of pre-effector cells. Additionally, cells predisposed toward the effector or memory fates are uniquely localized within the lymphoid organs during infection^43^. With the more pronounced alteration to the ratio of pre-effector to pre-memory cells in the complete absence of aPKC, the overabundance of pre-effector cells may have affected the consequent occupation of an appropriate niche, thereby altering necessary encounters with additional cytokines^41^ that could impact fate determination. In these ways, aPKC-deficient pre-effector cells, that might otherwise be predisposed toward the long-lived effector fate, may not have been optimally exposed to signals and cues that would promote their survival into and through the peak of the adaptive immune response.

Although differentiation of aPKC-deficient CD8^+^ T cells was increased toward the terminal effector fate, memory CD8^+^ T lymphocytes still formed. It is possible that these memory cells arose in a linear fashion directly from daughter cells predisposed toward the effector fates, perhaps through stochastic encounters with cytokines or other important fate determining signals. Alternatively, because complete loss of aPKC did not completely ablate asymmetric divisions or the generation of pre-memory first division cells, memory CD8^+^ T lymphocytes may have arisen from continued asymmetric divisions by a small number of aPKC-deficient cells. This may be a result of cell type-specific functions of aPKC, which may serve to maintain polarity as an activated CD8^+^ T cell divides^23^ rather than to establish polarity, as is the case in model organisms^24^,^25^,^26^. If aPKC is only active during mitosis, some proteins may already be polarized prior to mitosis and the absence of aPKC may only partially affect maintenance of this asymmetry through the completion of division. Because asymmetric division may require contact with an antigen-presenting cell^22^, it is also possible that stochastic interactions with an antigen-presenting cell during T cell division could allow proteins that function to establish and maintain a polarized immune synapse^44^ to maintain asymmetry during some divisions in the absence of aPKC. Alternatively, other polarity proteins and polarity networks^45^,^46^,^47^,^48^,^49^ may function to regulate asymmetry in CD8^+^ T cells and compensate in the absence of aPKC.

Our data suggest that aPKC plays a critical role only during the first CD8^+^ T lymphocyte division. Adoptive transfer of aPKC-deficient pre-effector or pre-memory first division cells at equal numbers revealed no intrinsic defect in these cells. Moreover, the phenotype caused by aPKC deficiency appeared to only have been a function of alterations in the differentiation patterns of effector and memory cells. With the continued decline of aPKC-deficient cells into the memory phase (i.e. following the death of terminal effector cells), any defects present early during the response were no longer apparent, indicating that loss of aPKC did not affect the functionality of long-lived effector and memory T lymphocytes. It is likely that aPKC-deficient pre-effector and pre-memory cells would still be exposed to important extrinsic signals^41^,^43^, allowing these precursor cells to progress into fully mature cell fates after the initial balance of effector- and memory-fated first division cells had been altered.

Unexpectedly, expression of KLRG1 and IL-7R at the peak of the immune response did not correspond to increased adoption of the terminal effector fate by aPKC-deficient cells. A lack of change in the percentage of aPKC-deficient CD8^+^ T lymphocytes expressing high levels of either putative marker at the peak of the immune response may indicate that certain aspects of CD8^+^ T lymphocyte differentiation and the generation of heterogeneous populations of cells may be “hardwired” into the immune response via cytokine signaling, metabolic processing, or other environmental cues. However, the findings that overexpression of IL-7R does not increase survival into the memory phase^50^ and that KLRG1^hi^ cells clearly survive into later stages of the immune response^4^,^5^,^23^ suggest that expression of these markers may not be suitable for delineating heterogeneity within the immune response or predicting cell death and survival. Indeed, examination of the gene expression profiles of individual IL-7R-expressing CD8^+^ T cells at 7 days post-infection has suggested that these cells may not be homogeneous, but instead may be a population comprised of fully mature terminal effector, effector memory, and central memory T cells^19^. These findings and our data suggest that alternative markers should be explored to more precisely study the heterogeneous populations of CD8^+^ T lymphocytes present during an adaptive immune response.

Generating diversity during an immune response is necessary for producing robust protection against infection and subsequent rechallenge^51^. The finding that activated CD8^+^ T lymphocytes can undergo asymmetric divisions at the initiation of the adaptive immune response^19^,^21^ provides a potential mechanism by which these diverse fates are specified. Here, we provide evidence that complete loss of the evolutionarily conserved polarity protein, aPKC, substantially reduces asymmetric divisions, thereby increasing the proportion of cells destined for the terminal effector lineage at the expense of the memory fates. Altering this initial balance of pre-effector to pre-memory first division cells increased differentiation into the terminal effector fate, thus indicating that asymmetric division plays a critical role in regulating early specification of effector and memory fates and the generation an optimal adaptive immune response.

## Methods

### Mice

All animal work was approved by the University of California, San Diego Institutional Animal Care and Use Committee and performed in accordance with the Institutional Animal Care and Use Guidelines. All mice were housed in specific pathogen-free conditions prior to use. *Prkcz*^fl/fl^*Prcki*^fl/fl^ mice were bred to *Cd4*^Cre^ mice. *Cd4*^Cre^*Prkcz*^fl/fl^*Prcki*^fl/fl^ mice were bred with OT-I TCR transgenic mice that recognize chicken OVA peptide SIINFEKL (residues 257–264)/K^b^. Wild-type C57/BL6J recipient mice were purchased from the Jackson Laboratory.

### CFSE labeling and *in vitro* cell culture

Splenocytes were isolated from OT-I mice and labeled with 5μM CFSE for 9 min at 37 °C. Reactions were quenched with FBS, and CD8^+^ T cells were isolated with a negative selection magnetic microbeads kit (Miltenyi Biotec), according to the manufacturer’s protocol. Splenocytes were harvested from wild-type mice and irradiated for 15 min at 3000 rads. T cells were depleted using magnetic microbeads (Miltenyi Biotec) and pulsed with 1μM OT-I peptide (SIINFEKL). Cells were cultured together for 3 days and analyzed on an Accuri C6 (BD Biosciences) with FlowJo software (Treestar).

### Adoptive cell transfers and infections

For primary infections, 5 × 10^3^ OT-I CD45.1^+^CD8^+^ T cells were adoptively transferred into wild-type CD45.2^+^ recipients, followed by infection intravenously one day later with 5 × 10^3^ CFU of *Listeria monocytogenes* expressing full-length chicken ovalbumin (Lm-OVA). Cell numbers for adoptive transfer experiments were measured and equalized between wild-type and aPKC-deficient T cells prior to transfer. Measurement of cell numbers was based on absolute cell counts from an Accuri C6 cytometer (BD Biosciences) and the percentage of CD8^+^ T lymphocytes within a donor mouse. Blood was collected from mice at 7, 14, 35, or 50 days post-infection in 5 mM EDTA solution. Blood samples were lysed with Red Cell Lysing Buffer (Sigma) for 15 minutes before staining for indicated markers. Splenocytes were isolated from recipient mice at 5, 7, or 50 days post-infection. For rechallenge experiments, 10^4^ memory OT-I CD45.1^+^CD8^+^ T cells were adoptively transferred into wild-type CD45.2^+^ recipients, followed by infection intravenously one day later with 10^5^ CFU of Lm-OVA. Blood was collected on days 5, 6, 7, and 8 post-rechallenge and analyzed as above. To isolate cells that had undergone their first division, 2 × 10^6^ OT-I CD8^+^ T cells were first labeled with CFSE, as above, prior to adoptive transfer, and splenocytes from recipient mice were harvested at 48 hours post-infection. Lymphocyte fate-tracking experiments were performed as previously described^19^. Briefly, 500 sorted IL-2Rα^hi^ or IL-2Rα^lo^ first division cells were adoptively transferred into infection-matched recipients. At day 50 post-transfer, recipients were rechallenged with 10^5^ CFU Lm-OVA, and population expansion was tracked in the blood, as above.

### T lymphocyte confocal microscopy

Immunofluorescence of T cells was performed as previously described^21^ with the following antibodies: anti-β-tubulin (T8328) (Sigma); anti-IL-2Rα (PC61.5), anti-T-bet (4B10) (eBioscience); anti-proteasome 20S C2 (ab3325) (Abcam); anti-IFN-γR (2E2) (Biolegend); anti-IRF4 (IRF4.3E4) (Biolegend); and anti-mouse Alexa Fluor 488, anti-rat Alexa Fluor 488, anti-mouse Alexa Fluor 647, anti-rabbit Alex Fluor 647, streptavidin Alexa Fluor 647, and anti-rat Alexa Fluor 647 (Life Technologies). DAPI (Life Technologies) was used to detect DNA. Cells undergoing cytokinesis were identified by dual nuclei and pronounced cytoplasmic cleft by brightfield. Acquisition of image stacks was performed at room temperature as previously described^21^ using a FV1000 laser scanning confocal microscopy system (Olympus) with an inverted microscope (IX81), a 40x/1.30NA oil objective, and FV10-ASW Viewer Software. The volume of 3D pixels (voxels) containing the designated protein fluorescence was quantified within each nascent daughter in cytokinetic cells as previously described^21^ using ImageJ software.

### Antibodies and flow cytometry

The following antibodies were used: T-bet (4B10), Eomes (Dan11mag), Bcl2 (BCL/10C4), IRF4 (IRF4.3E4), IL-2Rα (3C7), CD27 (LG.7F9), CD8a (53–6.7), CD45.1 (A20), CD62L (MEL-14), KLRG1 (2F1), IL-7R (A7R34), CD44 (1M7), Vα2 (B20.1), CD4 (RM4–5), IFN-γ (XMG1.2), TNFα (MP6-XT22), IL-2 (JES6-5H4) and F(ab′) 2 anti-rabbit anti-IgG and were obtained from Biolegend or eBioscience. Rabbit anti-Tcf-1 (C63D9) antibody was obtained from Cell Signaling Technology. Anti-human PE-conjugated Granzyme B (GB11) was obtained from Life Technologies. For intracellular staining of T-bet, Eomes, Bcl2, Tcf-1, IRF4, and Granzyme B, FoxP3/Transcription Factor Staining Buffer Kit was used (eBioscience). For intracellular detection of IFNγ, TNFα, and IL-2, CD8^+^ T cells were stimulated for 6 hrs at 37 °C *ex vivo* with 1μM OT-I peptide in the presence of brefeldin A (Sigma); cells were fixed in 4% paraformaldehyde (Electron Microscopy Services) and permeabilized before staining. All samples were analyzed on an Accuri C6 or FACSCanto (BD Biosciences) flow cytometer with FlowJo software (Treestar).

### BrdU pulse-chase and apoptosis assays

For BrdU pulse-chase experiments, mice were injected intraperitoneally with 1mg BrdU solution (BD Biosciences). After 1 hour, spleens were harvested, as described above, and cells were stained for BrdU using the BrdU flow kit obtained from BD Biosciences, according to the manufacturer’s protocol. Apoptosis was assessed following incubation with 7-AAD viability staining solution (Biolegend) for 15 minutes at room temperature or incubation with CellEvent Caspase-3/-7 Green Flow Cytometry Assay kit (eBioscience), according to manufacturer’s protocol. Mitochondrial membrane potential (Δψm) was assessed using Mitoflow (Cell Technology, Inc.), according to the manufacturer’s protocol.

## Additional Information

**How to cite this article**: Metz, P. J. *et al.* Regulation of Asymmetric Division by Atypical Protein Kinase C Influences Early Specification of CD8^+^ T Lymphocyte Fates. *Sci. Rep.* **6**, 19182; doi: 10.1038/srep19182 (2016).
